# Rapid molecular culture versus conventional culture for periprosthetic joint infection: diagnostic performance and clinical relevance

**DOI:** 10.5194/jbji-11-453-2026

**Published:** 2026-07-31

**Authors:** Elizabeth Morreel, Paul Savelkoul, Inge van Loo

**Affiliations:** 1 Department of Medical Microbiology, Infectious Diseases and Infection Prevention, Maastricht University Medical Centre, Maastricht, the Netherlands; 2 Care and Public Health Research Institute (CAPHRI), Maastricht University, Maastricht, the Netherlands

## Abstract

**Introduction**: Periprosthetic joint infection (PJI) remains a major complication of joint arthroplasty, requiring rapid and accurate pathogen identification to guide appropriate antimicrobial therapy. Conventional culture is limited by prolonged turnaround times and reduced sensitivity, particularly following antibiotic exposure. This study evaluated the diagnostic performance and clinical relevance of Molecular Culture^®^ (MC), a rapid PCR-based assay, using sonication fluid (SF) obtained during revision arthroplasty. **Methods**: SF samples from patients undergoing hip or knee revision arthroplasty (2019–2023) were retrospectively analysed. Patients were classified as PJI or non-PJI according to European Bone and Joint Infection Society criteria. MC results were compared with conventional SF culture. Positive percentage agreement (PPA) and negative percentage agreement (NPA) were calculated using culture as the reference standard. Clinical agreement was assessed using all available microbiological results and relevant clinical information, including recent antibiotic exposure. **Results**: A total of 203 SF samples from 172 patients were included. Analytical PPA and NPA of MC were 68.1 % (95 % CI, 59.9–75.3) and 84.6 % (95 % CI, 73.9–91.4), respectively. Following clinical correlation, PPA and NPA at patient level were 73.2 % (95 % CI, 64.9–80.2) and 95.6 % (95 % CI, 85.2–98.8), respectively. MC identified 11 additional clinically relevant pathogens and provided results within approximately 4 h. **Conclusions**: MC demonstrated high clinically adjusted agreement and faster turnaround times than conventional culture, supporting its role as an adjunctive diagnostic tool for PJI. Its value may be greatest in patients with uncertain infection status or prior antimicrobial exposure, where rapid exclusion of infection could facilitate earlier optimisation of antimicrobial therapy.

## 1 Introduction

As the number of joint replacement procedures continues to increase worldwide, complications such as periprosthetic joint infection (PJI) are becoming more prevalent (Patel, 2023). Now recognised as the leading cause of revision surgery (Dutch Arthroplasty Register, 2024), PJI poses a major challenge to both patient outcomes and healthcare systems. It is associated with significant morbidity, including prolonged antimicrobial treatment, impaired mobility, and psychological strain. In addition, PJI imposes a substantial economic burden, involving direct costs such as repeated surgeries and extended hospitalisation (Alt et al., 2025), as well as indirect costs related to prolonged rehabilitation, caregiver dependency, and loss of productivity (Sadoghi et al., 2025; Diep et al., 2025).

Timely diagnosis of PJI is critical for effective management and improved clinical outcomes. Distinguishing infected from non-infected cases is essential, as it informs both surgical and antimicrobial treatment strategies. In patients undergoing revision surgery, rapid microbiological diagnosis enables early initiation of targeted antimicrobial therapy, thereby reducing reliance on empirical broad-spectrum antibiotics. However, conventional culture, the current gold standard, has several important limitations. Although standard culture of aerobic and facultative anaerobic microorganisms is often detected within 24–48 h, final culture results in acute PJI may still require several days and in some cases up to 1 week. In chronic PJI, extended incubation periods for anaerobic and slow-growing microorganisms may be required for up to 2 weeks (Morreel et al., 2025; Talsma et al., 2021). This can lead to potential delays in treatment decisions. Furthermore, culture may fail to detect pathogens, particularly when the causative pathogen is present in low abundance or when patients have received prior antimicrobial therapy (Esteban et al., 2025).

Molecular diagnostic techniques that detect pathogen-specific DNA or RNA have gained increasing attention as complementary tools to conventional culture (Olearo et al., 2025). By bypassing the need for microbial growth, these methods can directly identify pathogens from clinical specimens, providing a valuable alternative when culture is slow, yields false-negative results, or is affected by prior antibiotic use. Consequently, molecular diagnostics can offer substantially faster turnaround times, facilitating earlier clinical decision-making and initiation of targeted therapy.

Despite their potential, the widespread implementation of molecular diagnostic techniques has been limited by several challenges, particularly high costs and analytical complexity. In recent years, multiplex PCR assays (Gardete-Hartmann et al., 2024b; Pascual et al., 2024; Malandain et al., 2018; Sigmund et al., 2019) have been used increasingly in the field of bone and joint infections. However, the diagnostic scope of multiplex PCR is inherently constrained by the predefined targets included in the panel. As a result, clinically relevant pathogens, including coagulase-negative staphylococci and *Cutibacterium acnes*, may not be detected.

To overcome these limitations, molecular diagnostic methods must balance broad-range pathogen detection and diagnostic accuracy with speed and practical feasibility. Molecular Culture^®^ (MC) represents one such approach. This PCR-based method is based on interspace profiling (IS-pro), targeting the 16S–23S ribosomal intergenic spacer region of bacteria. This region displays species-specific length and sequence polymorphisms, enabling accurate bacterial identification. In addition, MC allows detection of polymicrobial infections and, according to the manufacturer, provides results within 4 h.

In this study, we evaluated the diagnostic performance of Molecular Culture^®^ using sonication fluid specimens obtained during revision surgery. Sonication fluid is a practical and suitable specimen, as sufficient sample volume is usually available, and it represents the biofilm present on the prosthetic surface. We assessed whether this approach could match or outperform conventional culture in detection of clinically relevant pathogens, with a particular focus on turnaround time and clinical applicability.

## 2 Methods

### 2.1 Patients and samples

Sonication fluid samples from patients who underwent revision arthroplasty of the hip or knee between 2019 and 2023 were included. Implants were sonicated for 1 min at 40 kHz in sterile Ringer lactate using an ultrasonic bath (Bactosonic BS14, Bandelin GmbH, Berlin, Germany). Sonication fluid was cultured on aerobic and anaerobic solid media and in enrichment broth according to our previously validated protocol (Morreel et al., 2025), including incubation of enrichment media for up to 14 d. During routine clinical care, periprosthetic tissue specimens and, where available, synovial fluid samples were processed in parallel using the standard microbiological protocol as previously described. Only sonication fluid samples were stored at - 20 °C and were therefore available for subsequent Molecular Culture^®^ analysis. Data regarding concurrent microbiological culture results from all intraoperative specimen types, previous culture results, and prior antibiotic use were recorded. Retrospective research is exempt from Dutch law on medical research (WMO). Nevertheless, ethical approval was granted by the local medical ethics committee (METC 2023-0059). All data were de-identified, coded, and analysed anonymously.

### 2.2 Definitions

Sonication fluid (SF) samples were classified as originating from PJI or non-PJI cases according to European Bone and Joint Infection Society (EBJIS) diagnostic criteria (McNally et al., 2021). SF cultures with growth of > 50 CFU mL^−1^ were considered confirmatory for PJI. If growth in SF culture was < 50 CFU mL^−1^, but the same microorganism was found in > 1 intraoperative tissue(s) and/or fluids, this was also considered confirmatory for PJI. Additionally, other confirmatory criteria (e.g. clinical and blood workup) and the patient's clinical history, including microbiological findings from concurrent intraoperative tissue and synovial fluid culture results and previous culture results, were taken into account. Furthermore, the final diagnosis was determined by the multidisciplinary team (MDT), consisting of microbiologists and orthopaedic surgeons, when confirmatory criteria were not met. The non-PJI group therefore consists of patients who underwent aseptic revisions (no suspicion of infection) and patients in whom PJI was suspected, but cultures remained negative. Antibiotic use was recorded if the patient had received IV and/or oral antibiotics within 2 weeks prior to revision surgery.

### 2.3 Molecular Culture assay

**Figure 1:**
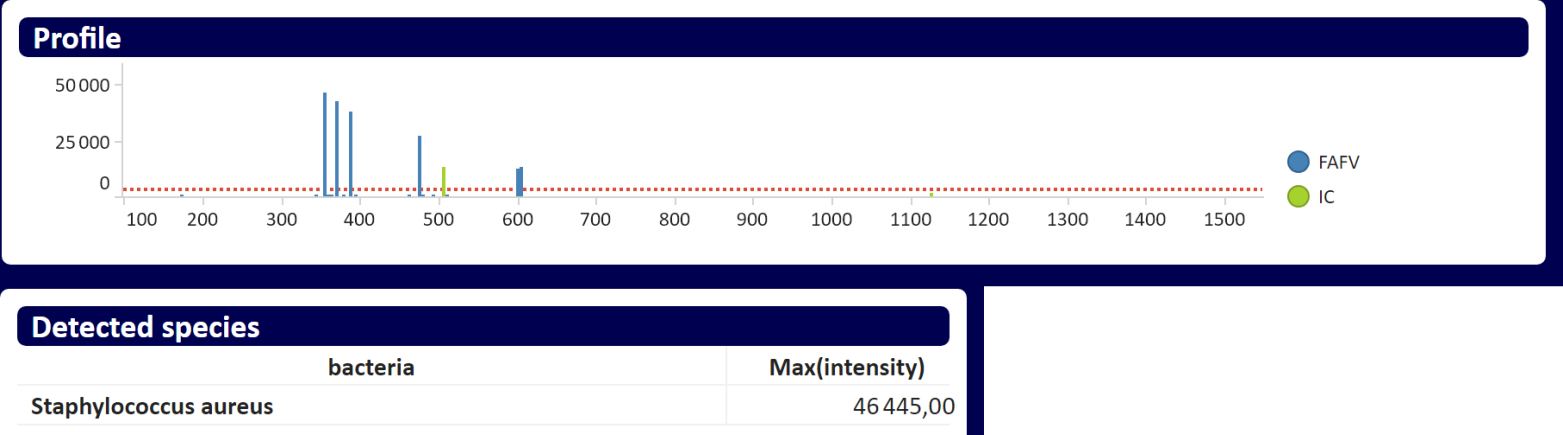
Example of a positive *Staphylococcus aureus* result as shown in the Antoni database: y axis – amplicon length (bp); x axis – signal intensity (RFU; relative fluorescence units); horizontal dotted red line – signal threshold; vertical blue lines – Firmicutes, Actinobacteria, Fusobacteria, Verrucomicrobia matched peaks; vertical green line – internal amplification control (IAC) matched peak.

A total of 200 µL of sample was combined with 250 µL bacterial shock buffer 1 (inbiome) and incubated at 95 °C for 10 min while shaking at 800 rpm. Subsequently, 25 µL of bacterial shock buffer 2 (inbiome) was added to the samples. Then, DNA extraction was performed on a MagNa Pure 96 system (Roche Diagnostics) using the DNA and Viral NA Small Volume Kit, with 200 µL of the pre-treated sample. Extracted DNA was eluted in a volume of 50 µL . Two PCRs were performed, each using 10 µL of sample DNA. The PCR primers were labelled with phylum-specific fluorophores. One PCR targets the phyla Firmicutes, Actinobacteria, Fusobacteria, Verrucomicrobia, and Bacteroidetes; the other PCR targets Proteobacteria and an internal amplification control (IAC). The final PCR products were combined and analysed for amplicon length fluorescence intensity (relative fluorescence units, RFU) on an ABI3500 fragment analyser (ThermoFisher). Analysis of ABI3500 output data was performed on Antoni, inbiome's lab cloud software. This software matches amplicon length to bacterial species using a dedicated database (inbiome). Samples yielding peaks above the cut-off intensities were classified as positive (Fig. 1). For certain microorganisms, species- or genus-level differentiation is not possible, as results are reported as predefined equivalence sets by the Antoni software. For *Cutibacterium* species, a genus often found in background signals, a threshold of > 10 000 RFU was considered clinically relevant, based on previous data according to the manufacturer (inbiome). Samples were classified as negative when peaks were below signal thresholds, but only if the IAC peaks were present.

### 2.4 Statistical analysis and data interpretation

Analytical positive percentage agreement (PPA) and negative percentage agreement (NPA) were calculated at the sample level by comparing Molecular Culture (MC) results with those obtained by culture. For culture-positive samples, MC was considered concordant only when identical pathogens were identified at the genus and species level. MC results were considered concordant when the microorganism identified by culture was included within the equivalence set reported by MC. In polymicrobial samples, concordance required detection by MC of all pathogens identified by culture. Any non-identical findings were classified as false-negative MC results.

For culture-negative samples, MC results were considered concordant only when no pathogen was detected. Detection of any pathogen by MC in culture-negative samples was classified as a false-positive result.

In addition, clinical PPA and NPA were calculated at the patient level by incorporating available clinical microbiological data of the same patient, including prior culture results and results from other specimen types, such as additional sonication fluid, periprosthetic tissue, and synovial fluid. 

## 3 Results

### 3.1 Study population and diagnostic characteristics

A total of 203 SF samples of 172 patients were included in this study, which included 145 confirmed PJI and 27 non-PJI cases. Demographic characteristics and classification criteria are summarised in Table 1. In 21 of the non-PJI cases, patients underwent revision surgery for reasons other than a suspected infection. In the six remaining cases, there was a clinical suspicion of PJI, based on previous culture results and/or clinical signs and blood workup. However, these cases could not be confirmed by EBJIS criteria (Table A1). A minority of patients ( n=16 , 9.3 %) had received antibiotics in the 2 weeks prior to revision surgery. In culture-positive PJI ( n=130 ), time to diagnosis ranged from 1 to 16 d (median: 3 d). By comparison, MC provided results within approximately 4 to 6 h after sample processing.

**Table 1:** Patient demographics and classification. PJI: periprosthetic joint infection; SF: sonication fluid.

	PJI ( n=145 )	Non-PJI ( n=27 )
Age, year, median (range)	70 (23–88)	70 (52–82)
Male, no. (%)	78 (53.8)	11 (40.7)
Site of arthroplasty– Hip – Knee	9748	189
Antibiotics within 2 weeks before surgery, no. (%)	13 (9.0)	3 (11.1)
PJI category, no. (%) – Early acute– Late acute– Late chronic	53 (36.5)43 (29.6)49 (33.8)	–––
SF culture-positive, no. (%)	130 (89.6)	–
Polymicrobial, no. (%)	6 (4.1)	–

### 3.2 Performance of Molecular Culture compared to conventional culture

#### 3.2.1 Analytical agreement

A total of 138 culture-positive samples and 65 culture-negative SF samples were included. Compared with conventional SF culture, analytical PPA and NPA were 68.1 % (95 % CI, 59.9–75.3) and 84.6 % (95 % CI, 73.9–91.4), respectively (Table A2). Among culture-positive samples, MC yielded identical pathogen identification in 94 samples (68.1 %). In 34 samples (31.9 %), MC produced a negative result despite a positive culture result. In 24 of these 34 samples, the culture load was low, defined as positivity in ≤ 2 enriched media. In addition, MC detected one or more non-corresponding pathogens in 10 samples, which were therefore classified as false-negative results at the analytical level (Tables 2, A4). Among culture-negative samples, MC yielded concordant negative results in 55 samples (84.6 %), whereas in the remaining 10 samples, MC detected one or more pathogens (Tables 2, A4).

**Table 2:** Additional pathogen detections by Molecular Culture with evidence for true positivity. Microorganisms are indicated in bold. SF: sonication fluid.

Confirmed PJI (microbiological diagnosis)	Result MC	Result SF culture	Additional clinical and laboratory information	Antibiotic use < 2 weeks pre-surgery
Yes	*Staphylococcus aureus* ***Streptococcus dysgalactiae***	*Staphylococcus aureus* (pathogen)		No
		*Streptococcus dysgalactiae* (contaminant^a^)		No
Yes	***Staphylococcus aureus*** *Pseudomonas aeruginosa* *Enterococcus faecalis*	*Pseudomonas aeruginosa* *Enterococcus faecalis*	Other samples, including additional SF sample: growth of *Staphylococcus aureus*	No
Yes	***Enterococcus faecalis***	*Staphylococcus warneri*	– Previous culture results: polymicrobial infection with *Enterococcus faecalis* amongst others – Other culture results: *Staphylococcus warneri*	Yes
Yes	***Streptococcus dysgalactiae***	Negative	Other samples, including additional SF sample: growth of *Streptococcus dysgalactiae*	No
Yes	***Streptococcus dysgalactiae***	Negative	Previous culture results elsewhere: *Streptococcus dysgalactiae*	Yes
Yes	***Streptococcus pneumoniae/mitis***	Negative^b^	Previous culture results elsewhere: *S. pneumoniae*	No
Yes	***Streptococcus dysgalactiae***	Negative	Previous culture results elsewhere: *Streptococcus dysgalactiae*	Yes
Yes	***Streptococcus dysgalactiae***	Negative	Previous culture results elsewhere: *Streptococcus dysgalactiae*	No
Yes	***Streptococcus dysgalactiae***	Negative	Other samples: growth of *Streptococcus dysgalactiae*	No
Yes	***Staphylococcus aureus***	Negative	Previous cultures results elsewhere: *Staphylococcus aureus*	No
No	***Streptococcus dysgalactiae***	Negative	Previous culture results elsewhere^c^: *Streptococcus dysgalactiae*	Yes

^a^ No other samples with growth of *Streptococcus dysgalactiae* and growth of < 50 CFU mL^−1^ in sonication fluid. ^b^ Contaminant growth: *Staphylococcus epidermidis* in 1 out of 2 enriched media. ^c^ From blood culture only; no positive intraoperative samples.

#### 3.2.2 Clinical agreement

When incorporating clinical information, PPA and NPA for culture-proven PJI at patient level were 73.2 % (95 % CI, 64.9–80.2) and 95.6 % (95 % CI, 85.2–98.8), respectively (Table A3). At the patient level, three of the mismatches in MC results were reclassified as true-positive findings (Table 2). Including patient data for the culture-negative samples showed supporting evidence for true positivity in 8 of these 10 samples (Table 2). Overall, MC identified 11 additional pathogens for which supporting clinical evidence for true positivity was available (Table 2). 

## 4 Discussion

The present study demonstrates that MC provides clinically relevant pathogen detection with substantially shorter turnaround time than conventional culture. When assessed analytically using culture as a reference standard, MC demonstrated moderate positive percentage agreement (68.1 %) and high negative percentage agreement (84.6 %). However, in routine clinical practice, SF culture results are not interpreted independently. Diagnostic criteria for PJI have historically varied, yet all definitions include concordant growth of the same microorganism in two or more intraoperative samples (McNally et al., 2021; Parvizi et al., 2018; Osmon et al., 2013). In line with clinical practice, a key strength of the present study is the integration of comprehensive clinical information alongside conventional culture results to assess the clinical relevance of MC findings. By integrating prior culture results, microbiological findings from other specimen types, and relevant clinical data such as recent antibiotic exposure, MC identified 11 additional pathogens with supporting evidence of true infection. Importantly, several cases initially classified as false-positive or false-negative at the analytical level were reclassified as clinically relevant following clinical correlation (Table 2). In four of these cases, recent antibiotic exposure may have contributed to culture negativity. Consequently, incorporation of clinical data improved both PPA and, in particular, NPA (73.2 % and 95.6 %, respectively). This high clinically adjusted NPA is particularly relevant in routine practice, where distinguishing low-grade infection from aseptic failure is often challenging. As a result, patients are often treated empirically with broad-spectrum antibiotics until infection has been excluded. In this context, MC offers a means to rapidly rule out infection, allowing earlier discontinuation or avoidance of unnecessary antimicrobial therapy. Given that culture-based exclusion of infection may require up to 14 d, the short turnaround time of MC represents a clear advantage, particularly for slow-growing organisms such as *Cutibacterium* spp.

Several limitations should be considered. First, MC relies on equivalence sets within its identification database. In this study, results were considered concordant when the cultured microorganism belonged to the equivalence set identified by MC. However, such ambiguity may significantly influence empirical antibiotic choices. For example, differentiation between *Klebsiella* spp. and *Enterobacter* spp. is clinically relevant in our epidemiological setting, as empirical therapy would differ. In addition, MC does not provide antimicrobial susceptibility data. While conventional culture enables targeted therapy through susceptibility testing, reliance on MC alone could result in overtreatment or inappropriate therapy. This limitation is partly mitigated by adjustment of the empirical therapy based on local epidemiology but remains an important consideration for clinical implementation. False-negative MC results were observed. No consistent pattern was identified with respect to the microorganisms missed or the bacterial load, suggesting that technical factors may have contributed. Potential explanations include suboptimal DNA extraction efficiency due to sample dilution, as substantially smaller volumes are used for MC compared with culture. Furthermore, prolonged sample storage and pre-analytical factors related to sample handling may have affected overall specimen quality and DNA integrity. Finally, interpretation challenges are particularly relevant for microorganisms that may represent either true pathogens or contamination. *Cutibacterium* spp. are a typical example, as they are established causes of PJI but are also frequently encountered as contaminants. Although a signal threshold of ≥ 10 000 RFU was applied in this study, no uniform thresholds exist for many other organisms. Consequently, low-level contamination remains an inherent limitation of sensitive molecular techniques. Similar challenges have been described in other studies evaluating molecular methods for bone and joint infections (Olearo et al., 2025; Hong et al., 2023), where variability between techniques and the absence of standardised cut-offs complicate interpretation. Our findings reinforce the importance of clinical context and the continued need to evaluate multiple specimens and sample types to distinguish true infection from contamination.

Despite these limitations, MC demonstrated a high clinical agreement, supporting its role as a screening tool, particularly in cases where the likelihood of PJI is uncertain. Its rapid turnaround time enables earlier clinical decision-making and may facilitate timely de-escalation of empirical antimicrobial therapy. MC has been validated for use with synovial fluid and tissues, thereby enabling application across the full range of specimen types used in PJI diagnostics (Bos et al., 2023; Gardete-Hartmann et al., 2024a). It may be particularly valuable in patients who have received prior antimicrobial therapy, as certain organisms, such as streptococci, are particularly susceptible to antibiotics and therefore more likely to yield culture-negative results. Several molecular approaches have been investigated for the diagnosis of PJI, each with distinct advantages and limitations. Targeted multiplex PCR platforms offer rapid pathogen identification but are inherently dependent on the microorganisms included in the predefined panel. This limitation can be addressed by expanding target coverage or by applying broad-range approaches, such as 16S ribosomal RNA PCR or next-generation sequencing (NGS), which have demonstrated the ability to detect organisms missed by conventional culture and targeted molecular assays (Olearo et al., 2025). However, these sequencing-based approaches often require additional bioinformatic processing, specialised expertise, and longer analytical workflows, which may limit routine clinical implementation. MC may offer a balance between highly targeted assays and sequencing-based methods by combining a panbacterial approach and rapid turnaround time with a workflow that does not require complex bioinformatic analysis. Further comparative studies are required to determine the relative diagnostic performance and clinical utility of these different molecular strategies. From an implementation perspective, the relatively simple workflow of MC may facilitate integration into routine laboratory practice, making it more accessible than sequencing-based approaches. Future strategies such as sample pooling and higher-throughput testing may further improve cost-effectiveness.

In conclusion, MC provides rapid and clinically relevant information for the diagnosis of PJI, particularly in cases where infection needs to be excluded or where prior antibiotic exposure limits the sensitivity of conventional culture. Its clinical value lies in its use as a complementary tool, requiring careful interpretation within the clinical context. In contrast to approaches that position molecular diagnostics as a rescue test, our findings support early integration of MC in the diagnostic pathway. By enabling prompt exclusion of infection, early MC testing may reduce unnecessary empirical (intravenous) antibiotic use, shorten hospitalisation, and decrease healthcare costs. From an antimicrobial stewardship perspective, this strategy has the potential to optimise both diagnostic efficiency and downstream clinical management.

## Data Availability

The data generated and used for analysis during the current study are publicly available in the Dutch Archiving and Networked Services (DANS) repository (Data Station Life Sciences) at 10.17026/LS/ME8ZHV (Morreel, 2026).

## Appendix A

**Table A1:** Suspicion of PJI (periprosthetic joint infection), non-confirmed: suggestive criteria.

Case no.	Clinical and blood workup	Radiological signs of prosthesis loosening	Previous (intraoperative or preoperative) culture results positive	Antibiotic use < 2 weeks pre-surgery
1	History of recent bacteremia, C-reactive protein (CRP) > 10 mg dL^−1^	No	Blood culture and synovial fluid culture: *Escherichia coli*	Yes
2	No	Yes	No	Yes
3	History of recent fever, CRP > 10 mg dL^−1^, history of recurring erysipelas	No^*^	Synovial fluid culture: *beta-hemolytic streptococci*	Yes
4	No	Yes	No	No
5	CRP > 10 mg dL^−1^, previous wound-healing problems	No	No	No
6	History of recent bacteremia, CRP > 10 mg dL^−1^	No	Blood culture: *Streptococcus dysgalactiae*	No

^*^ No white blood cell scintigraphy performed, but positive PET-CT scan results were available.

**Table A2:** Contingency table analytical agreement at sample level.

	Conventional culture			
	(comparative method)			
	Positive	Negative		Total
Molecular Culture (candidate method)				
Positive	94	10		104
Negative	44	55		99
Total	138	65		203

**Table A3:** Contingency table clinical agreement at patient level.

	Conventional culture			
	(comparative method)			
	Positive	Negative		Total
Molecular Culture (candidate method)				
Positive	93	2		95
Negative	34	43		77
Total	127	45		172

**Table A4:** Additional pathogen detections by Molecular Culture with insufficient evidence for true positivity.

Confirmed PJI	Result MC	Result culture	Additional clinical and laboratory information	AB use < 2 weeks pre-surgery
No	*Streptococcus bovis/intermedius group*	Negative^a^	Other samples: no growth	No
No	*Streptococcus dysgalactiae*	Negative	Clinical history of recurrent erysipelas Previous cultures^b^: *Staphylococcus aureus,* *Streptococcus agalactiae* (group B)	Yes
Yes	*Streptococcus pneumoniae/mitis*	Negative	Other samples: no growth Previous culture results: *Staphylococcus aureus*	Yes
Yes	*Staphylococcus aureus*	*Staphylococcus epidermidis*	Other samples: growth of *Staphylococcus epidermidis* only	No
Yes	*Escherichia coli*	*Staphylococcus aureus*	Other samples: growth of *Staphylococcus aureus *only	No
Yes	*Streptococcus agalactiae* (group B) *Streptococcus bovis group*	*Streptococcus agalactiae* (group B)	Other samples: growth of *Streptococcus* group B only	No
Yes	*Streptococcus dysgalactiae*	*Streptococcus dysgalactiae* *Escherichia coli*	Other samples: growth of *Streptococcus dysgalactiae* and *Escherichia coli*	No
Yes	*Streptococcus agalactiae* (group B)	*Cutibacterium avidum*	Other samples: growth of *Cutibacterium avidum* only	No
Yes	*Cutibacterium acnes*	*Abiotrophia defectiva*	Other samples: growth of *Cutibacterium acnes* only; MALDI-TOF score of *Abiotrophia defectiva* 99.9 % in SF sample	No

^a^ Contaminant growth: Cutibacterium acnes and *S. sanguinis* in 1 out of 2 enriched media. *S. sanguinis* does not belong to equivalence set of Streptococcus bovis/intermedius group. ^b^ Previous intraoperative samples of fracture-related infection. Note that MALDI-TOF denotes matrix-assisted laser desorption/ionization time-of-flight mass spectrometry.
